# Simplifying Oral health evaluation: a novel approach through single-item surveys

**DOI:** 10.1186/s12903-023-03794-2

**Published:** 2024-06-07

**Authors:** Takashi Zaitsu, Tomoya Saito, Akiko Oshiro, Yoko Kawaguchi, Ichiro Kawachi

**Affiliations:** 1 Department of Oral Health Promotion, Graduate School of Medical and Dental Sciences, Tokyo Medical and Dental University, 1-5-45 Yushima, Bunkyo-ku, Tokyo, 113-8549 Japan; 2 Department of Social and Behavioral Sciences, Harvard T.H. Chan School of Public Health, 677 Huntington Ave., 7th Floor, Boston, MA 02115 USA

**Keywords:** Self-rated oral health, Japanese, Validity, Oral health behavior, Decayed teeth, Missing teeth, Periodontitis, Gingivitis, Oral hygiene

## Abstract

**Background:**

This study adopts a novel approach of using single-item surveys to simplify the assessment of oral health status and behaviors among Japanese private sector employees. We aimed to establish the validity of self-reported oral health in relation to clinical dental examinations, and to elucidate the relationship between oral diseases, health behaviors, and self-assessments. A secondary aim was to explore the association of self-rated oral health with oral health behaviors.

**Materials and methods:**

Self-administered questionnaires and dental examinations were obtained from 2262 Japanese private sector employees. Workers self-rated their overall oral health status according to five choices: “very good,” “good,” “fair,” “poor,” or “bad.” Self-reports were then compared with the results of clinical dental examinations, which included measuring the oral hygiene index (DI-S), the number of decayed teeth, periodontal status (Community Periodontal Index) and number of missing teeth. Convergent validity was also tested by examining the correlations of self-reported oral health status with oral health behaviors.

**Results:**

Overall, 30.8% of workers reported their oral health as “poor” or “bad.” “Poor” or “bad” oral health status was significantly correlated with missing teeth, periodontitis, and decayed teeth. However, lower correlations were found for gingivitis and the oral hygiene index. Most self-reported oral health behaviors were correlated with self-rated oral health; exceptions were “tooth brushing instructions received in a dental clinic,” “having a primary-care dentist,” and “habitual snacking between meals.”

**Conclusions:**

Self-rated oral health provides reasonably valid data, and correlated well with clinically assessed oral health status, including dental caries, periodontal status, and tooth loss. Convergent validity was also found for oral health behaviors.

**Trial registration:**

Clinical trial registration number: UMIN000023011 (UMIN-CTR).

Date of clinical trial registration: 06/07/2016.

**Supplementary Information:**

The online version contains supplementary material available at 10.1186/s12903-023-03794-2.

## Introduction

Maintenance of good oral health is increasingly recognized as a factor in employee wellness and productivity [1]. However, dental examinations in the workplace require an investment in personnel, facilities, time, and expense. Questionnaire surveys could potentially provide a quick method to screen employees, and indeed epidemiologic surveys have widely adopted self-reported oral health measures because of their convenience [2]. However, the practical utility of self-reports depends on their validity.

Previous studies of the validity of oral health-related questionnaires have assessed correlations with clinical examinations [3], including number of remaining teeth and presence of dentures [4–6].

However, the oral health self-assessment questionnaires used to date have comprised many questions and have been time-consuming to complete [7, 8]. Reliability, validity, and simplicity are important for these questionnaires.

Among oral diseases, dental caries, periodontal diseases, and tooth loss have a high prevalence [9]. All of these diseases are treatable in a dental clinic setting [10]. Therefore, it is of clinical significance to identify these dental diseases in a simple questionnaire, which can be easily completed in a workplace context (such as annual health checkups).

The aim of this study was to investigate whether a simple questionnaire could detect dental diseases. We tested the reproducibility and validity of self-rated oral health related to dental caries, periodontal disease, tooth loss, oral hygiene, and oral health behavior in adults.

The purposes of this study were:

1. To conduct a single questionnaire survey on adults’ oral health and investigate whether it reflects the actual oral health status.
2. To evaluate how the convergent validity of self-reported oral health status through correlations with oral health maintenance behaviors.

## Materials and methods

### Study participants

The survey was conducted in 2015, and the sampling frame consisted of 2262 workers employed in private sector 36 Japanese companies. The companies included in this study were selected after explaining the research objectives to each, resulting in participation from 36 companies across the nation. In terms of company size, the composition was as follows: 14 companies with fewer than 50 employees, 9 companies with 50 to 99 employees, 9 companies with 100 to 299 employees, and 4 companies with 300 to 999 employees. The industry categories were classified according to the Japan Standard Industrial Classification (October 2013): Construction had 2 companies with 54 employees, Manufacturing comprised 14 companies with 850 employees, Electricity, Gas, Heat Supply, and Waterworks involved 4 companies with 152 employees, Transportation and Postal Services included 5 companies with 345 employees, Wholesale and Retail Trade encompassed 4 companies with 349 employees, Accommodation and Food Services had 1 company with 26 employees, Education and Learning Support consisted of 2 companies with 322 employees, Medical Care and Welfare included 1 company with 61 employees, and the Service Industry was represented by 3 companies with 103 employees. There were 2262 participants at first who consented to participate in the study. Participants completed a self-administered questionnaire and underwent an oral examination at their companies. The final sample used for analysis consisted of 2262 workers (1721 men and 541 women; aged 18–72 years; mean age: 42.6 ± 11.7 years) who provided complete data. The Research Ethics Committee, Faculty of Dentistry, Tokyo Medical and Dental University (No. 1152) approved the study protocol. Prior to enrolling in the study, the individuals were provided with a detailed explanation of the entire research protocol and had signed the informed consent document.

### Questionnaires

Each subject completed a self-administered questionnaire containing items on self-rated oral health and oral health behavior prior to the dental examination. The content of the questionnaire were as follows:

1. Self-rated oral health

The question evaluating self-rated oral health was: “How do you think about your current oral health condition?” Response options were: 1. Very good; 2. Good; 3. Fair; 4. Poor; 5. Bad.

(2) Oral health behavior

Of the 20 items in ‘Lifelong Teeth Support Program’ [11], the following 10 items were included in the questionnaire: (1) Brushing teeth before bed; (2) Brushing teeth at the workplace; (3) Use of an interdental brush or dental floss; (4) Use of fluoridated toothpaste; (5) Tooth brushing instructions received in a dental clinic; (6) Making time to go to a dental clinic; (7) Having a primary-care dentist; (8) Undergoing dental examination at least once a year; (9) Habitual snacking between meals; and (10) Smoking habits.

### Oral health status

Oral examinations with visual and tactile techniques were performed to assess the oral health status (dental, periodontal, and oral hygiene status) using World Health Organization (WHO) periodontal probes and mouth mirror. Dental status was assessed by the number of decayed teeth (DT: Dental caries) and missing teeth (MT: Tooth loss). Also, periodontal status was assessed with the Community Periodontal Index (CPI) [12]. CPI divided the dentition into six parts and scored each of the six parts for gum bleeding (gingivitis) (code 0: healthy; code 1: gingival bleeding) and periodontal pockets (periodontitis) (code 0: healthy; code 1: periodontal pocket depth of 4–5 mm; and code 2: periodontal pocket depth of 6 mm or more). We calculated the number of bleeding sites (bleeding code 1) and the periodontal pocket depth of 4 mm or more (periodontal pocket code 1–2). In addition, the study excluded participants with code X (missing index tooth) in any of the six parts. The simplified debris index (DI-S) component of the simplified oral hygiene index (OHI-S) was used to assess dental plaque (oral hygiene) [13–15]. There were a total of five dental examiners involved in our study. To ensure calibration, we conducted a training session for all participating dentists. During this session, we thoroughly explained and distributed standardized guidelines for oral health assessment. Following this, we carried out a calibration process based on these guidelines to ensure that all dentists applied a consistent set of evaluation criteria.

### Data analysis

We performed a one-way analysis of variance (ANOVA) with the objectively assessed oral health status as the dependent variable and self-rated oral health status as the independent variable. Analysis of covariance (ANCOVA) was conducted to calculate oral health status indexes with the self-rated oral health as the independent variables, adjusting for age, gender. Crude logistic-regression analyses were performed using self-rated oral health (0: very good–good; 1: fair–bad) as the dependent variable, and oral health status and oral health behaviors as the independent variables. The statistical package for the Social Sciences (SPSS) version 23.0 (International Business Machines, Tokyo, Japan) was used for statistical analysis, and the significance level was set at *p* < 0.05.

## Results

### Relationship between self-rated Oral health, clinical Oral health status, and demographic factors

The results of oral health status by age and gender are presented in Table 1. The results indicated that younger individuals, both males and females, had a higher incidence of decayed teeth. The presence of missing teeth and periodontitis, indicated by pockets deeper than 4 mm, tended to be higher in the old age group. No age-related trends were observed for gingival bleeding or oral cleanliness as measured by the Debris Index-Simplified (DI-S). In all cases, males exhibited worse trends compared to females.

**Table 1:** Distribution of oral health status by gender and age group

			Decayed teeth		Missing teeth		No. of bleeding		No. of PD > = 4 mm		DI-S	
		N	Mean	SD	Mean	SD	Mean	SD	Mean	SD	Mean	SD
Male	−29	292	1.07	2.26	0.16	0.59	2.43	2.07	0.24	0.72	0.60	0.49
	30–39	380	0.89	1.94	0.42	1.41	2.16	2.04	0.37	0.99	0.52	0.48
	40–49	469	0.90	1.97	0.87	1.88	1.90	1.92	0.44	1.01	0.48	0.42
	50-	580	0.86	2.15	2.84	4.34	2.16	1.97	0.81	1.31	0.56	0.50
	Total	1721	0.91	2.08	1.31	3.01	2.13	2.00	0.51	1.10	0.54	0.48
Female	−29	98	0.50	1.35	0.28	0.77	1.65	1.85	0.09	0.38	0.34	0.33
	30–39	132	0.49	1.40	0.36	1.00	1.32	1.74	0.20	0.69	0.34	0.38
	40–49	180	0.46	1.07	0.68	1.27	1.49	1.66	0.39	1.04	0.32	0.30
	50-	131	0.40	0.91	1.86	2.76	1.75	1.68	0.63	1.12	0.31	0.29
	Total	541	0.46	1.17	0.82	1.76	1.54	1.72	0.35	0.92	0.33	0.32

**Fig. 1:**
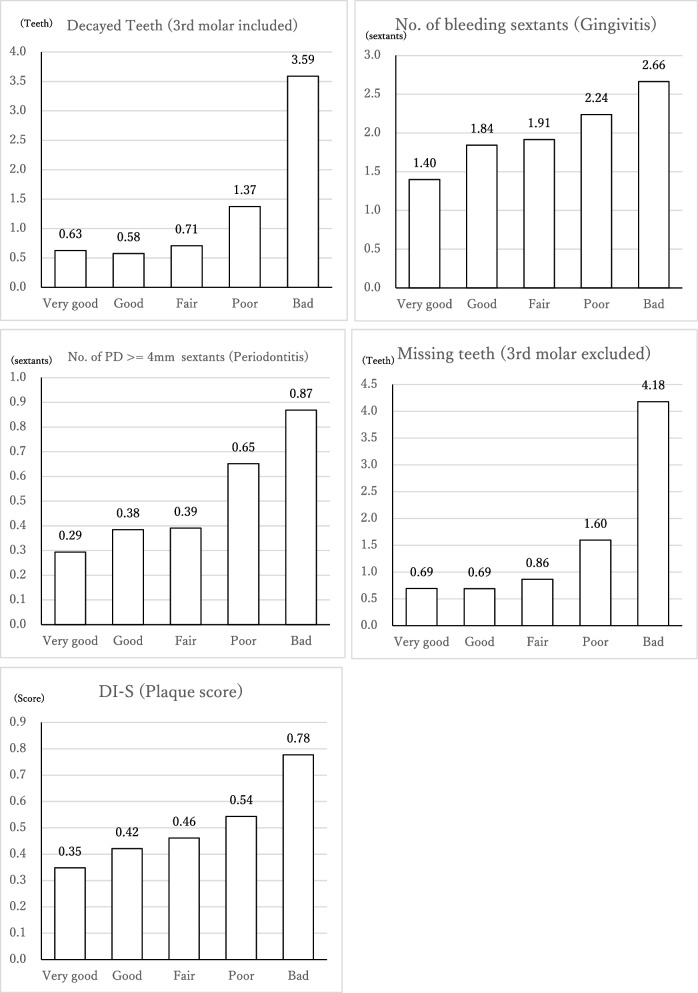
“Q How is the health condition of your teeth and gum” VS oral health status (continuous number)

Self-rated oral health responses were “very good” in 6.8%, “good” in 13.2%, “fair” in 49.2%, “poor” in 25.4%, and “bad” in 5.4%. The correlation between self-rated oral health and clinical assessed oral health status is shown in Fig. 1. Dental plaque and gingivitis showed a linear trend with worse self-assessment of oral health. Dental caries, periodontitis, and tooth loss were similarly distributed in the three groups of “very good,” “good,” and “fair” oral health in self-rated oral health, with an inflection point in objective oral health status when self-assessments were “poor” or “bad”. ANOVA revealed that dental caries, periodontitis, and tooth loss were not significantly different between “very good,” “good,” and “fair” self-ratings, while “poor” and “bad” were significantly different from the other response items. Furthermore, ANCOVA adjusted for age and gender revealed significant differences in all oral health statuses. Particularly, those who self-rated their oral health as ‘very good’ showed significantly better conditions in all aspects of oral health. Conversely, individuals who rated their oral health as ‘bad’ exhibited significantly worse conditions in both gingivitis and periodontitis (Table 2).

**Table 2:**
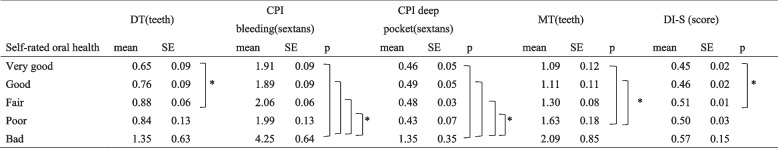
Oral health status according to self-rated oral health (ANCOVA)

Adjusted by age and gender

*p < 0.05

### Associations between poor/bad self-rated Oral health and Oral health status/behaviors: a crude logistic regression analysis

For all oral health status, significant correlations were seen with the self-rating of oral health (Table 3). Additionally, for 7 of the 10 oral health behaviors, significant correlations were seen with the self-rating of oral health (Table 3). “Poor” and “bad” self-rated oral health were significantly associated with poor oral health behaviors related to: (1) Brushing teeth before bed; (2) Brushing teeth at the workplace; (3) Use of an interdental brush or dental floss; (4) Use of fluoridated toothpaste; (6) Making time to go to a dental clinic; (8) Undergoing dental examination at least once a year; and (10) Smoking habits. There were no significant differences in the oral health behaviors related to: (5) Tooth brushing instructions received in a dental clinic; (7) Having a primary-care dentist; and (9) Habitual snacking between meals.

**Table 3:** Oral health status and behaviors associated with poor/ bad self-rated oral health using the crude logistic regression analysis

		Odds ratio	95% CI.for odds ratio		Sig.
			Lower	Upper	
Oral Health Status					
(1) Decayed Teeth (3rd molar included)	0	reference			
	1-	1.749	1.391	2.199	<.001
(2) Bleeding (Gingivitis)	No	reference			
	Yes	1.279	1.032	1.586	0.024
(3) PD ≧4 mm (Periodontitis)	No	reference			
	Yes	1.297	1.003	1.676	0.047
(4) Missing Teeth (3rd molar included)	0	reference			
	1-	2.177	1.714	2.765	<.001
(5) DI-S (Plaque score)	< 0.5	reference			
	≧0.5	1.502	1.213	1.858	<.001
Oral health behaviors					
(1) Tooth brushing before sleeping	Daily	reference			
	Sometimes	2.048	1.630	2.572	<.001
	Never	1.372	.994	1.894	.054
(2) Brushing teeth in the workplace	Daily	reference			
	Sometimes	1.925	1.462	2.534	<.001
	Never	1.690	1.321	2.163	<.001
(3) Use of an interdental brush or dental floss	Daily	reference			
	Sometimes	1.078	.794	1.463	.632
	Never	1.520	1.139	2.029	.004
(4) Use fluoride toothpaste	Yes	reference			
	No	1.524	1.214	1.914	<.001
	Don’t Know	1.423	1.154	1.754	.001
(5) Receiving tooth brushing instructions in a dental clinic	Yes	reference			
	No	1.166	.970	1.402	.103
(6) Making time to go to a dental clinic	Yes	reference			
	No	2.369	1.974	2.843	<.001
(7) Having a primary-care dentist	Yes	reference			
	No	1.187	.986	1.429	.070
(8) Undergoing dental examinations at least once a year in a dental clinic	Yes	reference			
	No	2.031	1.645	2.506	<.001
(9) Habitual eating between meals	Never	reference			
	Sometimes	.935	.729	1.200	.599
	Daily	1.242	.935	1.649	.134
(10) Smoking habits	No	reference			
	Past	1.271	.912	1.771	.157
	Yes	1.865	1.529	2.275	<.001

## Discussion

The one-item oral self-rated health assessment has been widely used in population-based oral epidemiology studies. However, validation studies have remained sparse, and to our knowledge, this is the first study to clinically validate the instrument in the context of employee screening in the workplace. Our results provide moderate support for the validity of the simple, one-item instrument in this context.

In particular, we found that dental plaque (oral hygiene) and gingival bleeding, which are the early indicators of dental diseases, deteriorated monotonically as the self-evaluation of oral health status worsened. On the other hand, conditions requiring dental treatment, such as dental caries, periodontitis, and loss of teeth, were similar among individuals self-assessing their oral health as “very good,” “good,” or “fair”. The prevalence of these more serious conditions increased sharply only among individuals who rated their oral health as “poor.”

Previous studies found that dental caries has a significant impact on patients’ oral health-related quality of life as well as self-assessments [16]. As the prevalence of untreated dental caries was low in our study, it is possible that many subjects considered that they were “fair (not good)” in view of their lack of dental caries.

Periodontal disease has been described as a silent disease [17, 18], and it has been reported that early-stage periodontal diseases are difficult to recognize. However, a study reported that in severe periodontal disease (periodontitis), such as deep periodontal pockets and loss of attachment, there were significantly more respondents self-reporting that they had a poor quality of life or poor oral health [1, 16, 19]. Periodontal pockets of 4 mm or more were more likely to affect individual ratings of their oral health status as being “poor”. This was also the case with tooth loss. The mean number of MT was fewer than 1 in all patients with “very good,” “good,” and “fair” self-assessments.

Meanwhile, gingivitis, which is the early phase of periodontal disease, showed a different trend from dental caries and periodontitis. It did not exhibit a steep curve, and the stages were clearly different even between “good” and “fair” self-assessments. This was also the case with the oral hygiene status (DI-S), where we showed that the people who perceived their oral health as “good” and those who perceived it as “fair” differed in their oral hygiene status.

Dental diseases in the initial stage can be improved by self-care, while serious oral conditions necessitate a treatment [20, 21]. Based on the results of our study, serious dental disease should be demarcated at the cut-point between “very good/good/fair” versus “poor/bad.” In identifying severe dental disease, it is important to identify respondents who self-assess their oral health status as “poor/bad”. In the analysis adjusted for age and gender, it was found that individuals who self-rated their oral health as bad had significantly worse periodontal health, whether in terms of gingivitis or periodontitis. This suggests that a poor self-assessment of oral health could considerably increase the risk of periodontal disease.

Turning to the associations between self-rated oral health and oral health behaviors, we found that most poor behaviors were also associated with worse self-rated oral health (“poor” or “bad”). Individuals with poor subjective symptoms in their oral cavity actually had poor oral health. Our study suggests that a simple questionnaire was effective in detecting both oral health status and oral health behaviors. Previous questionnaire surveys assessing the oral health had many questionnaire items or only targeted specific diseases and were less versatile [7, 22]. However, our questionnaire could be used in any setting, such as general medical examination.

Our study has several limitations. Since it was conducted among workers employed in companies rather than in the general Japanese population, our results may not be generalizable. Moreover, there have been reports indicating that oral health status varies according to industry categories and gender-based occupational classifications within companies. This suggests a need for further research to meticulously examine the unique characteristics of each company to develop a more comprehensive understanding of these variations [23, 24]. Additionally, in this study, periodontal disease was assessed using CPI. Normally, a more accurate evaluation of periodontal disease would involve assessing attachment levels or conducting detailed periodontal examinations. However, due to the brief duration of surveys conducted within the companies, we opted for the simplified approach of using CPI. Also, this study has limitations regarding the number of oral health status items assessed, not including aspects such as tooth pain, denture pain, and malocclusion. This is due to our focus on caries, periodontal diseases, and tooth loss, which are more commonly observed in workers. Furthermore, as this study adopts a novel approach of using a single-item survey to assess oral health status and behaviors, it was not able to elucidate the factors affecting self-assessed oral health and the extent of their impact. We also investigated oral diseases in relation to dental caries, periodontal disease, tooth loss, and oral hygiene status. However, future studies should validate self-reports against other oral diseases, such as oral mucosal diseases, temporomandibular joint disorders and halitosis.

Furthermore, future studies should also focus on Health Literacy. Health Literacy significantly affects both the accuracy of self-assessment and oral health behaviors. Higher Oral Health Literacy (OHL) likely contributes to more accurate self-assessments and better oral health practices. Conversely, individuals with lower OHL may lack the necessary knowledge or understanding to maintain proper oral health and make informed decisions, potentially leading to poorer oral health habits and clinical outcomes. It is considered essential to conduct future surveys focusing on Health Literacy.

## Conclusion

Those who self-rated their oral health as “poor” or “bad” were found to be particularly at risk for dental diseases based on clinical examination, as well as poor oral health behaviors. In environments where it is difficult to conduct dental checkups, it may be possible to identify high-risk individuals through this questionnaire.

### Supplementary Information

**Additional file 1.**

## Data Availability

The corresponding author will provide the data utilized and examined in this study upon a reasonable request.

## Abbreviations

DI-S: Simplified debris index
OHI-S: Simplified oral hygiene index
WHO: World Health Organization
DT: Decayed teeth
MT: Missing teeth
CPI: Community Periodontal Index
ANOVA: Analysis of variance
ANCOVA: Analysis of covariance
SPSS: Statistical Package for the Social Sciences

## References

[1] Batista MJ, Perianes LB, Hilgert JB, Hugo FN, Sousa ML. The impacts of oral health on quality of life in working adults. Braz Oral Res. 2014;28:0040. https://www.scielo.br/j/bor/a/y7SBZMkBrhdq4LHxWdML4sy/?lang=en. 10.1590/1807-3107bor-2014.vol28.0040 25166762

[2] Safdar N, Abbo LM, Knobloch MJ, Seo SK (2016). Research methods in healthcare epidemiology: survey and qualitative research. Infect Control Hosp Epidemiol.

[3] Palmqvist S, Söderfeldt B, Arnbjerg D (1991). Self-assessment of dental conditions: validity of a questionnaire. Community Dent Oral Epidemiol.

[4] Matsui D, Yamamoto T, Nishigaki M (2016). Validity of self-reported number of teeth and oral health variables. BMC Oral Health.

[5] Ueno M, Zaitsu T, Shinada K, Ohara S, Kawaguchi Y (2010). Validity of the self-reported number of natural teeth in Japanese adults. J Investig Clin Dent.

[6] Pitiphat W, Garcia RI, Douglass CW, Joshipura KJ (2002). Validation of self-reported oral health measures. J Public Health Dent Spring.

[7] Balappanavar A, Sardana V, Nagesh L, Ankola A, Kakodkar P, Hebbal M (2011). Questionnaire *vs* clinical surveys: the right choice?-a cross-sectional comparative study. Indian J Dent Res.

[8] Robinson PG, Nadanovsky P, Sheiham A (1998). Can questionnaires replace clinical surveys to assess dental treatment needs of adults?. J Public Health Dent Summer.

[9] Kim JK, Baker LA, Davarian S, Crimmins E. Oral health problems and mortality. J Dent Sci. 2013;8(2) 10.1016/j.jds.2012.1012.1011. 10.1016/j.jds.2012.12.011 PMC3885153 24416472

[10] Devaraj C, Eswar P (2012). Reasons for use and non-use of dental services among people visiting a dental college hospital in India: a descriptive cross-sectional study. Eur J Dent.

[11] Japan Dental Association, Lifelong Teeth Support Program. 2013; https://www.jda.or.jp/dentist/program/pdf/ph_01.pdf. Accessed Feb 24, 2019.

[12] World Health Organization (2013). Oral health surveys: basic methods.

[13] Durovic E, Markovska N, Martinukova B, Mincik J, Sutak J (1986). Correlation of the Loe-Silness GI and the green-Vermillion OHI-S. Prakt Zubn Lek.

[14] Erickson JD (1973). Statistical tests for the OHI-S and PI: a commentary. J Dent Res.

[15] Moore BJ (1977). Measuring treatment and scale bias effects by linear regression in the analysis of OHI-S scores. J Dent Res.

[16] Haag DG, Peres KG, Balasubramanian M, Brennan DS (2017). Oral conditions and health-related quality of life: a systematic review. J Dent Res.

[17] Tonetti MS, Jepsen S, Jin L, Otomo-Corgel J (2017). Impact of the global burden of periodontal diseases on health, nutrition and wellbeing of mankind: a call for global action. J Clin Periodontol.

[18] Nazir MA (2017). Prevalence of periodontal disease, its association with systemic diseases and prevention. Int J Health Sci.

[19] Allen PF (2003). Assessment of oral health related quality of life. Health Qual Life Outcomes.

[20] Jönsson B, Lindberg P, Oscarson N, Öhrn K (2006). Improved compliance and self-care in patients with periodontitis–a randomized control trial. Int J Dent Hyg.

[21] Van Der Weijden F, Slot DE (2011). Oral hygiene in the prevention of periodontal diseases: the evidence. Periodontol.

[22] Locker D, Allen PF (2002). Developing short-form measures of oral health-related quality of life. J Public Health Dent.

[23] Irie K, Tsuneishi M, Saijo M, Suzuki C, Yamamoto T (2022). Occupational difference in Oral health status and behaviors in Japanese workers: a literature review. Int J Environ Res Public Health.

[24] Harada Y, Takeuchi K, Furuta M, Tanaka A, Tanaka S, Wada N, Yamashita Y (2018). Gender-dependent associations between occupational status and untreated caries in Japanese adults. Ind Health.

