# Supplementary material for: Simplifying Oral health evaluation: a novel approach through single-item surveys
Source: BMC Oral Health. 2024 Jun 7;24:669. doi: 10.1186/s12903-023-03794-2 (PMC11161979; doi:10.1186/s12903-023-03794-2)
Supplement: Supplementary file 1 — Additional file 1. [file 12903_2023_3794_MOESM1_ESM.docx]

Appendix Figure A “Q How is the health condition of your teeth and gum” VS oral health status (categorical)
